# Are adolescents’ physical activity and body-related factors associated with medically attended injuries?

**DOI:** 10.3389/fped.2022.901011

**Published:** 2022-11-01

**Authors:** Viktoryia Karchynskaya, Jaroslava Kopcakova, Andrea Madarasova Geckova, Peter Bakalár, Andrea F. de Winter, Sijmen A. Reijneveld

**Affiliations:** ^1^ Department of Health Psychology and Research Methodology, Faculty of Medicine, P. J. Safarik University in Kosice, Kosice, Slovakia; ^2^ Department of Community & Occupational Medicine, University Medical Center Groningen, University of Groningen, Groningen, Netherlands; ^3^ Institute of Applied Psychology, Faculty of Social and Economic Sciences, Comenius University in Bratislava, Bratislava, Slovakia; ^4^ Department of Sports Educology and Humanistics, Faculty of Sports, University of Presov, Presov, Slovakia

**Keywords:** adolescents, medically attended injuries, physical activity, body composition, cardiovascular fitness

## Abstract

**Background:**

Injuries are the major cause of disability and death during adolescence, representing a significant public health burden among youth. Body-related factors such as body composition and cardiovascular fitness (CVF) may affect adolescents’ vulnerability to injuries. As evidence is lacking, we aimed to explore the associations of medically attended injuries with adolescents’ physical activity (PA) and body-related factors, and whether these associations are modified by age, gender and family affluence.

**Methods:**

We used data on 888 11- to 15-year-old adolescents (mean age = 13.5, 56% boys) from the Health Behaviour in School-aged Children study conducted in 2018 in Slovakia. We used binary logistic regression analysis to assess the association of medically attended injuries with adolescents’ PA and body-related factors (body composition, CVF), considering age, gender and family affluence.

**Results:**

Adolescents were more likely to report medically attended injuries if they were physically very active (odds ratio/confidence interval OR/CI:2.76/1.83–4.15) or active (OR/CI:1.91/1.27–2.87) rather than inactive. Body-related factors were not associated with medically attended injuries among adolescents. Moreover, age, gender and family affluence did not modify the association of medically attended injuries with adolescents’ PA and body-related factors. The only exception was the modifying effect of gender: the association of medically attended injuries with being very active was stronger in boys (OR/CI: 3.04/1.32–6.99).

**Conclusion:**

Very physically active adolescent boys are the most vulnerable group of adolescents in terms of injuries. PA promotion programmes should further consider gender-specific strategies aimed at preventing injuries.

## Introduction

Injuries are the major cause of disability and death during adolescence (1), representing a significant public health burden among the young population (2). According to the results of the Health Behavior in School-Aged Children (HBSC) study, between 2010 and 2018, there has been an alarming increase in the number of medically attended injuries among Slovak adolescents, i.e., injuries requiring treatment in a doctor's office or health clinic (3). Inchley et al. (1) reported that the prevalence of medically attended injuries among Slovak adolescents was 48.7%. Importantly, this prevalence was higher than the mean prevalence in the 45 countries and regions participating in the 2017/2018 HBSC study and also higher than the results of the 2009/2010 and 2013/2014 HBSC studies in Slovakia (4). As Davison et al. (5) noted, sports and recreational activities are the main cause of injury among adolescents. However, it is well known that physical activity (PA) has a great impact on health in childhood and adolescence (6) and can help improve cardiorespiratory and muscular fitness (7), as well as bone health (8). On the other hand, PA also has adverse effects, such as a risk of injuries, with some requiring medical attention (9, 10). Moreover, participation in organised sports in adolescence is an important risk factor for hospitalisation due to injures (11). This suggests that since injuries are the leading cause of disability and death among adolescents, injury prevention should be a public health priority with regard to adolescence.

Body-related factors, such as overweight/obesity or poor cardiovascular fitness (CVF, i.e., level of motor skills), may make adolescents more vulnerable to injuries. Richmond et al. (12) found that the risk of sports injury in obese adolescents was higher than in adolescents with normal weight. Conversely, Warsh et al. (13) and Ezzat et al. (14) did not find evidence of a modifying effect of body composition on injury risk among adolescents. Furthermore, Carter and Micheli (15) and Watson et al. (16) noted that well-developed CVF might protect youth from future injury. However, Martin-Diener et al. (17) found that Swiss children with high levels of motor skills had an increased retrospective injury risk compared to children with normal levels. Moseid et al. (18) found that rapid increases in training load resulted in more injuries. In addition, previous studies did not show a relationship between CVF and injury risk level among adolescents (19–21). In other words, it is unclear whether body-related factors affect the risk of injury among adolescents.

The impact of adolescents’ PA and body-related factors on medically attended injuries may further depend on age, gender and family affluence issues. Regarding age, Inchley et al. (1) noted that younger adolescents have the highest injury risk of all age groups and carry the highest health burden. Regarding gender, boys experienced more sports injuries than girls did (22). However, a study of Schneider et al. (23) did not find gender differences in injury risk. In addition, as noted by Elgar et al. (24), family affluence is a potential determinant of injury among adolescents. According to Simpson et al. (25), lower family affluence was associated with increased risk for hospitalised and fighting injury; higher family affluence was associated with increased risks for sport injury. However, to date little research has examined these interactions.

Therefore, the aim of this study was to explore the associations of medically attended injuries with adolescents’ PA and body-related factors, and whether these associations are modified by age, gender and family affluence.

## Materials and methods

### Sample and procedure

We used data on 888 adolescents (mean age = 13.5, 56% boys) from the Health Behaviour in School-aged Children (HBSC) study conducted in 2018 in Slovakia. The HBSC is an international school survey conducted in collaboration with the World Health Organization to study the health and health-related behaviour of 11-, 13- and 15-year-old school children in their social context (26). We used a three-step sampling to obtain a representative sample. In the first step, 140 larger and smaller elementary schools located in rural as well as in urban areas from all regions of Slovakia were asked to participate. These were randomly selected from a list of all eligible schools in Slovakia obtained from the Slovak Institute of Information and Prognosis for Education. The school response rate (RR) was 77.9%. In the second step, we obtained data from 8,405 adolescents from the fifth to ninth grades of elementary schools in Slovakia, which have as target group adolescents aged 11- to 15-years. Student RR in the selected schools was 60.1%. Students were randomly selected in the line with the methodology and protocol of HBSC study. In the third step, 10% of elementary schools were randomly selected from the total sample of the HBSC study for anthropometric measurements (body height, body weight, body composition), and student RR was 81.8%. After cleaning the database of questionnaire errors (unspecified gender, age, etc.), our study sample regarded 888 adolescents aged 11–15 years.

The study was approved by the Ethics Committee of the Medical Faculty at P. J. Safarik University in Kosice (16N/2017). Parents were informed about the study *via* the school administration and could opt out if they disagreed with their child's participation. Participation in the study was fully voluntary and anonymous, with no explicit incentives provided for participation.

### Measures

*Medically attended injuries* were assessed by the single-item HBSC question asking: “During the past 12 months, how many times were you injured and had to be treated by a doctor or nurse?” with five possible answers ranging from “I was not injured in the past 12 months” to “4 times or more” (27). We dichotomised the answers into two categories—“no injuries” and “one or more injuries” (28).

We measured PA as a composite variable *adolescents’ PA* based on the combination of moderate-to-vigorous PA (MVPA) and engagement in organized sports. The combined PA index has been used in previous studies and has shown appropriate validity (29, 30). MVPA was measured by an item asking adolescents about the number of days over the past week that they were physically active for a total of at least 60 min per day (27). The engagement in organized sports regarded items dealing with team sports and individual sports, respectively, and was measured by asking adolescents: “In your leisure time, do you do any of these organized activities?” with response categories “yes” and “no” (27). The three categories of adolescents’ PA were: (1) inactive: adolescents who were active less than 5 days per week and were not engaged neither in team, nor in individual organised sports, (2) active: active 5–7 days per week or engaged in team or individual organised sports, and (3) very active: active 5–7 days per week and also engaged in team or individual organised sports.

We used body composition and cardiovascular fitness to measure body-related factors. *Body composition* was measured using body fat percentage (%) as determined by Bioimpedance Body Composition Analysis (BIA) with an InBody 230 device (Biospace Co., Ltd.). This marker has previously been used to measure body adiposity in children and adolescents in other studies (31, 32). Moreover, in a previous study by Karelis et al. (33) were indicated that the portable InBody 230 may be an acceptable device for measuring body fat percentage. The analysis was carried out according to the manufacturer's instructions (34). Adolescents were instructed prior to measurement to dress in a t-shirt and trousers or skirt. The starting weight was set to −0.5 kg, considering that we weighed the adolescents in their underwear. Boys and girls with a proportion of body fat of over 25% and 30% were considered to be overweight and obese, respectively (35, 36). We dichotomised body composition into two categories—“normal weight”, and—“overweight/obesity” (combination of overweight and obesity).

The level of *CVF* was assessed by the Ruffier index (RI) calculated from the measured values of pulse frequency at rest before the Ruffier test (P0), after performing 30 squats for 45 s under the sound of a metronome (P1) and after 1 min of rest in sitting (P2). Based on Sartor et al. (37) we modified the Ruffier test in the method of measuring the pulse rate (we replaced the original palpation measurement with measurements using SUUNTO DUAL pulse rate monitors) and in the length of physical and mental rest before the test (from the original 30 to 3 min of sitting for time reasons). We substituted the measured values of the pulse frequency into the formula: RI = ((P0 + P1 + P2)-200)/10. In addition, we used the CVF categories (i.e., excellent, good, below average, very poor) from Moravec (38), who created such standards based on population testing. Based on Fardman et al. (39), we dichotomised the level of CVF into two categories—“sufficient” (included “excellent”, “good”, “average”, and “below average” levels), and—“insufficient” (included “very poor” level).

*Demographic data* (age, gender) were collected using the following HBSC questions: “Are you a boy or a girl?” and “What year and month were you born?” (27). We dichotomised age into two categories—“younger” (11- and 12-years-old), and—“older” (from 13- to 15-years-old).

*Family affluence* was measured using the Family Affluence Scale III (FAS-III), which represents adolescents’ relative socioeconomic status in their respective country and is considered the gold standard to assess cross-national as well as national comparisons in the HBSC study and its methodology (1). FAS-III consists of six questions: “Does your family own a car, van or truck?” (No/Yes, one/Yes, two or more), “Do you have your own bedroom for yourself?” (Yes/No), “How many computers does your family own?” (None/One/Two/More than two), “How many bathrooms (room with a bath/shower or both) are in your home?” (None/One/Two/More than two), “Does your family have a dishwasher at home?” (Yes/No), “How many times did you and your family travel out of your country for a holiday/vacation last year?” (Not at all/Once/Twice/More than twice). In addition, the sum score of the six items showed high test-retest reliability (*r* = 0.90) and consistency between child and parent report (*r* = 0.80) (27). We computed the sum score, which we converted to a final score ranging from 0 to 1. We then created tertile categories of low (0–0.333), medium (0.334–0.666) and high (0.667–1) socioeconomic position (40).

### Statistical analyses

First, we described the sample using descriptive statistics. Next, we assessed the association of medically attended injuries (dependent variable) with adolescents’ PA, body composition and cardiovascular fitness (independent variables) using binary logistic regression leading to odds ratios (ORs) and 95% confidence intervals (CI), assessing crude associations (Model 1) and associations adjusted for age, gender and FAS (Model 2). Finally, we assessed whether age, gender and FAS modified the associations of medically attended injuries (dependent variable) with adolescents’ PA, body composition and cardiovascular fitness (independent variables) by adding two-way interactions with age, gender and FAS to the models. All analyses were performed using IBM SPSS Statistics 21 for Windows.

## Results

### Background characteristics

Of the respondents, 49.4% had medically attended injuries at least once or more per 12 months, 37.0% were physically very active, 22.7% were overweight or obese and 74.4% had a sufficient level of CVF. Of the respondents 61.1% were in the older age group (13–15 years old), 56.0% were boys and 44.2% had high family affluence (Table 1).

**Table 1:** Characteristics of the sample (*N* = 888, 11–15-years-old Slovak school-aged children, data collected in 2018).

Characteristic	*N* (%)
Medically attended injuries	
No injuries	417 (50.6)
1 or more injuries	407 (49.4)
Adolescents’ physical activity (PA)	
Very active (5–7 days MVPA and OLTA)	318 (37.0)
Active (5–7 days MVPA or OLTA)	311 (36.2)
Inactive (less than 5 days MVPA and no OLTA)	230 (26.8)
Body composition	
Normal weight	686 (77.3)
Overweight and obesity	202 (22.7)
Cardiovascular fitness (CVF)	
Sufficient	661 (74.4)
Insufficient	227 (25.6)
Age	
Younger (11–12 years old)	345 (38.9)
Older (13–15 years old)	543 (61.1)
Gender	
Boys	497 (56.0)
Girls	391 (44.0)
Family affluence	
Low	174 (25.7)
Middle	204 (30.1)
High	300 (44.2)

MVPA, moderate-to-vigorous physical activity; OLTA, organized leisure-time activities; numbers of missing cases per variables: medically attended injuries, 64; adolescents’ PA, 29; body composition, 0; CVF, 0; age, 0; gender, 0; family affluence, 210.

### The association of medically attended injuries with adolescents’ PA and body-related factors

Results of binary logistic regression (Table 2, Model 1) showed that adolescents were more likely to report medically attended injuries if they were physically very active or active in comparison to adolescents who were inactive. Body composition was not associated with medically attended injuries among adolescents. Furthermore, age and gender were not associated with medically attended injuries. However, adolescents who had middle or high family affluence were more likely to report medically attended injuries. Repeating the analysis with the ridit of the FAS-score instead of a categorized FAS-score yielded similar findings for the various associations (not shown). Moreover, CVF was associated with medically attended injuries, i.e., adolescents were less likely to report medically attended injuries, if they had an insufficient level of CVF. However, this effect was not significant anymore when confounding effect of age, gender and FAS was included (Table 2, Model 2).

**Table 2:** Association of medically attended injuries with adolescents’ physical activity, body composition, cardiovascular fitness, and socio-demographic factors: odds ratios (OR) and 95% confidence intervals (95%-CI), resulting from binary logistic regression models, crude and adjusted for age, gender and family affluence.

	Model 1 (crude) OR (95% CI)	Model 2 (adjusted) OR (95% CI)
Adolescents’ physical activity (PA)		
Very active (5–7 days MVPA and OLTA)	2.70 (1.88–3.87)***	2.76 (1.83–4.15)***
Active (5–7 days MVPA or OLTA)	2.06 (1.44–2.95)***	1.91 (1.27–2.87)**
Inactive (less than 5 days MVPA and no OLTA)	1 (Ref.)	1 (Ref.)
Body composition		
Overweight/obesity	0.74 (0.53–1.03)	0.72 (0.50–1.04)
Normal weight	1 (Ref.)	1 (Ref.)
Cardiovascular fitness (CVF)		
Insufficient	0.72 (0.52–0.98)*	0.77 (0.54–1.10)
Sufficient	1 (Ref.)	1 (Ref.)
Age		
Younger (11–12 years old)	1.01 (0.76–1.34)	-
Older (13–15 years old)	1 (Ref.)	
Gender		
Boys	1.30 (0.99–1.71)	-
Girls	1 (Ref.)	
Family affluence		
High	1.79 (1.22–2.61)**	-
Middle	1.58 (1.05–2.38)*	
Low	1 (Ref.)	

MVPA, moderate-to-vigorous physical activity; OLTA, organised leisure-time activities; Ref., reference category.

* *p* < 0.05.

** *p* < 0.01.

*** *p* < 0.001.

### The modifying effect of age, gender and family affluence on association of medically attended injuries with adolescents’ PA and body-related factors

**Figure 1:**
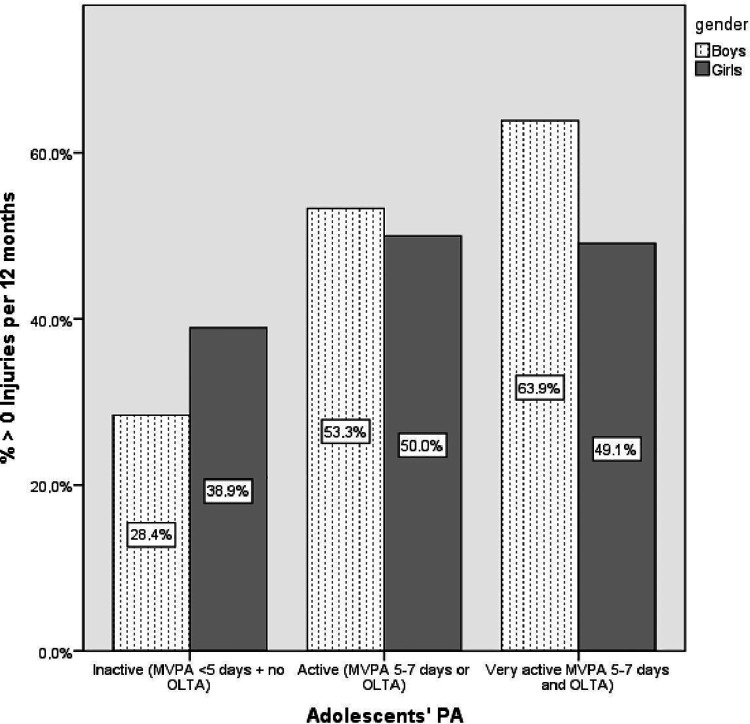
Moderation by gender of the association of medically attended injuries with adolescents’ physical activity. MVPA, moderate-to-vigorous physical activity; OLTA, organised leisure-time activities.

Age, gender and family affluence did not modify the association of medically attended injuries with adolescents’ PA and body-related factors (findings not shown in Table 2). The only exception was a modifying effect of gender on the association of medically attended injuries with adolescents’ PA. The association of medically attended injuries with being very active was stronger in boys (OR/CI: 3.04/1.32–6.99), see Figure 1.

## Discussion

This study explored the associations of medically attended injuries with adolescents’ PA and body-related factors in 888 Slovak adolescents from 11 to 15 years old. We found that adolescents were more likely to report medically attended injuries if they were physically very active or active rather than inactive and that this association was stronger among boys.

It is important to note that our composite variable for adolescents’ PA included engagement in organised sports. This aligns with the findings of Jespersen et al. (41) and Räisänen et al. (42) showing that injury prevalence was higher in sports club activities than in other leisure-time PA. In addition, Räisänen et al. (43) found that injuries were associated with higher frequency and intensity of PA, and these findings are also in line with our study. This indicates that very physically active adolescents can be classified as the most vulnerable group of adolescents in terms of injuries.

Unlike PA, body-related factors were not associated with medically attended injuries among adolescents. This aligns with the findings of Warsh et al. (13) and Ezzat et al. (14) showing no significant association of body composition with injury prevalence among adolescents. An explanation may regard the level of engagement in PA among adolescents; e.g., overweight or obese adolescents who lack the skill and confidence or have an insufficient CVF may not be able to cope with some sports situations. That may result in them deliberately avoiding situations that require physical effort, and thus they experience no injuries during PA because they have no PA (44). Therefore, body-related factors may have a protective effect on injury risk among adolescents. This evidently requires further study, as avoiding intensive PA is not desirable from the point of view of health promotion.

We found that gender modified the association of medically attended injuries with adolescents’ PA: the association of medically attended injuries with being very active was stronger in boys. Previous studies (22, 42, 43) have shown gender to be associated with injuries; e.g., boys had significantly more injuries than girls. An explanation might be the type of PA and way of practicing which boys and girls prefer; i.e., boys prefer more contact sports, behave more aggressively towards their opponents and take more risks (45). Moreover, boys might do sport more excessively than girls, so they may also be at higher risk of injury. The findings of Knowles et al. (46) and Schneider et al. (23) showed that after adjusting for the type and level of PA, gender differences in injury risk disappeared. In other words, gender and injuries are associated, and this may be due to the type of sports and the way of practicing that adolescents prefer.

In our study, the modifying effect of age and FAS on the association of medically attended injuries with adolescents’ PA and body-related factors was not significant. Räisänen et al. (43) reported that sport is one possible factor contributing to the differences in prevalence of injuries between age groups. In addition, Warsh et al. (13) found that adolescents reported a higher relative odds of being injured due to PA if they were in higher grades (9–10, i.e., 14–16 years), and had a middle or high family affluence. The ambiguity of research results in this area indicates that the putative contribution of these socio-demographic factors in relation to medically attended injuries among adolescents should further be studied.

## Strengths and limitations

The major strengths of this study regard its large, nationally representative sample of adolescents and the comparability of our data with the international data within the HBSC study. Another potential strength is the use body composition and cardiovascular fitness, which represent objective measurements (using body fat percentage and the Ruffier index).

Some limitations should also be mentioned. First, adolescents’ PA and medically attended injuries were measured by self-report, making them prone to reporting bias. This is unlikely to explain the associations found, but may have added some measurement error. Another limitation was the cross-sectional design of this study, which hinders conclusive inferences about causality. Therefore, our findings need to be confirmed in longitudinal studies.

## Implications

The results of this study can help inform intervention and prevention efforts to counteract the documented increase of injuries among adolescents. Our finding that adolescents with a higher level of PA were more likely to report medically attended injuries requires additional attention. In this case, for people involved in organised sports it is very relevant to be aware of this risk, to develop a culture of safety and to incorporate safety into the training of trainers and make adolescents aware of safety. Based on our findings about the moderating effect of gender, it seems that PA promotion programmes may further need to consider gender-specific strategies aimed at preventing injuries, e.g., when organizing sports teams for boys, injury prevention programmes should be provided to players-beginners, when they start playing in competitive divisions.

## Conclusion

Adolescents are more likely to report medically attended injuries if they have a higher PA. Body-related factors are not associated with medically attended injuries. Very physically active adolescent boys can be considered as the most vulnerable group of adolescents in terms of injuries.

## Data Availability

The raw data supporting the conclusions of this article will be made available by the authors, without undue reservation.
